# The full-length mitochondrial genome of *Tachysurus fulvidraco* (Siluriformes: Bagridae) from Geum river in Korea

**DOI:** 10.1080/23802359.2020.1831996

**Published:** 2020-10-27

**Authors:** Ha-Youn Song, Keun-Yong Kim, Keun-Sik Kim, Seung-Yong Kim, Hyoung-Joo Jeon

**Affiliations:** a Inland Fisheries Research Institute, National Institute of Fisheries Science, Gapyeong, Republic of Korea; b AquaGenTech Co., Ltd., Busan, Republic of Korea; c Research Center for Endangered Species, National Institute of Ecology, Yeongyang, Republic of Korea

**Keywords:** Mitogenome, Bagridae, *Tachysurus fulvidraco*, phylogenetic analysis

## Abstract

The full-length mitochondrial genome of the yellow catfish, *Tachysurus fulvidraco* was analyzed by the primer walking method. Its assembled mitochondrial genome was found to be 16,527 bp, consisting of 37 genes (13 protein-coding genes, 22 tRNA gens, and 2 rRNA gens). The gene content and order of *T. fulvidraco* were congruent with those of typical vertebrate fishes. In the phylogenetic tree, it showed the closet relationship to the another conspecific specimen from China and *Pseudobagrus koreanus* and well separated from the other species in the family Bagridae.

The species in the family Bagridae including *Tachysurus fulvidraco* are one of the economically important food fish of inland fisheries in Korea (Park and Lee 1996). However, the wild populations of bagrid species are suspected to be in decline owing environmental pollution and overfishing. In this study, the full-length mitogenome (mitochondrial genome) of *T. fulvidraco* was determined to provide the baseline data for the management of inland fishery resources.

A specimen of *T. fulvidraco* used in this study were collected from Geum River in South Korea in October 2019 (36°17′22.67″ N, 127°00′38.54″ E). The voucher specimen (NFRDI-FI-TS-0054112) was deposited in the collection of the Inland Fisheries Research Institute in South Korea. Its genomic DNA was extracted from the pelvic fin according to Asahida et al. (1996). Its mitogenome was amplified through two independent and overlapping PCR amplifications, and the PCR products were sequencing 26 sequencing primers by the primer walking method.

The mitogenome of *T. fulvidraco* was a circular molecule of 16,527 bp in total length (GenBank accession number, MT876426), consisting of 13 protein-coding genes, 2 ribosomal RNA (rRNA) genes, and 22 transfer RNA (tRNA) genes, and a control region. Its gene order was identical to those of typical vertebrates or bagrid species (Huang et al. 2016). The nucleotide matrix was generated from the first, second and third bases of each codon of 12 protein-coding genes, excluding *nad6*, 2 rRNA genes, and 22 tRNA genes. Phylogenetic analysis was conducted using RAxML 7.0.4 (Stamatakis 2006) for maximum likelihood (ML) analysis.

In this study, we analyzed the full-length mitogenome of *T. fulvidraco* from Geum River in South Korea and reconstructed the molecular phylogenetic tree to verify their taxonomic conclusion. In the phylogenetic tree, *T. fulvidraco* from Geum River in Korea grouped formed a monophyletic group with the another conspecific specimen from China and the two specimens clustered with *P. koreanus* (Figure 1). Ng and Kottelat (2007) re-described the taxonomic review on the species in the genera *Pseudogargrus* and *Tachysurus*, and transferred it to the genus *Tachysurus* based on morphological characteristics. In contrast, Chae et al. (2019) classified it as a species in the genus *Pseudobagrus*, *Pseudobagrus fulvidraco*. Our phylogenetic analysis supported its taxonomic position in *Pseudogargrus* by showing the closest phylogenetic relationship to the congeneric *P. koreanus*. In the future study, the taxonomic status of *T. fulvidraco* should be reappraised based on detailed morphological and molecular phylogenetic studies. Our complete mitogenome reported here will provide a useful baseline data for the inland fisheries management of dagrid fishes in South.

**Figure 1.**
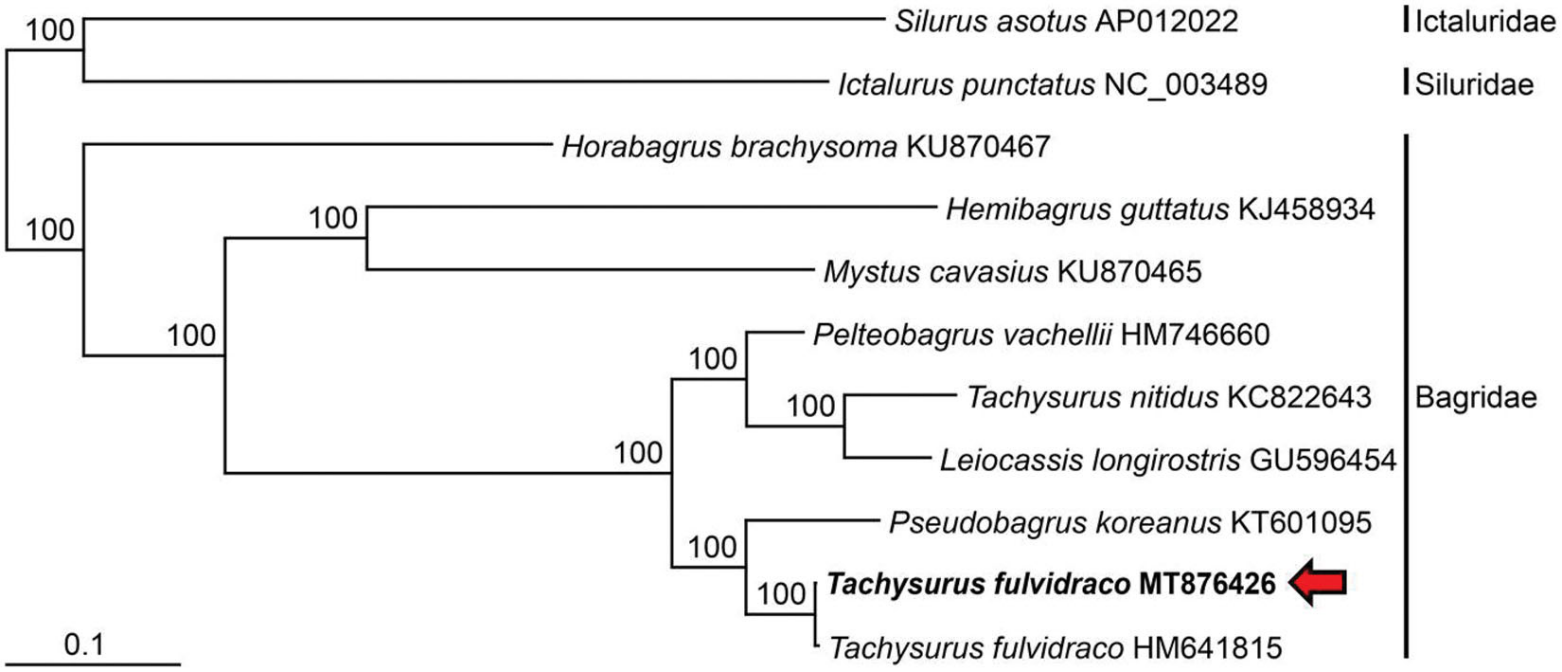
Maximum-likelihood (ML) phylogeny based on the full-length mitochondrial genomes from the *Tachysurus fulvidraco* (MT876426) belong to the order Siluriformes. The nucleotide sequence matrix included the codon positions of the 12 protein-coding genes. A bootstrap value above 50% in the ML analysis is indicated at each node. *Tachysurus fulvidraco* analyzed in this study is shown in red arrow.

## Data Availability

The data that support the findings of this study are openly available in NCBI web site [http://www.ncbi.nlm.nih.gov], reference number [MT876426]
